# The impact of voluntary exercise on relative telomere length in a rat model of developmental stress

**DOI:** 10.1186/1756-0500-5-697

**Published:** 2012-12-27

**Authors:** Martmari Botha, Laurian Grace, Kishor Bugarith, Vivienne A Russell, Martin Kidd, Soraya Seedat, Sian MJ Hemmings

**Affiliations:** 1 Department of Biomedical Sciences, Faculty of Medicine and Health Sciences, Stellenbosch University, Tygerberg, South Africa; 2 Department of Human Biology, University of Cape Town, Cape Town, South Africa; 3 Centre for Statistical Consultation, Stellenbosch University, Stellenbosch, South Africa; 4 Department of Psychiatry, Faculty of Medicine and Health Sciences, Stellenbosch University, Tygerberg, South Africa

**Keywords:** Stress, Exercise, Relative telomere length

## Abstract

**Background:**

Exposure to early adverse events can result in the development of later psychopathology, and is often associated with cognitive impairment. This may be due to accelerated cell aging, which can be catalogued by attritioned telomeres. Exercise enhances neurogenesis and has been proposed to buffer the effect of psychological stress on telomere length. This study aimed to investigate the impact of early developmental stress and voluntary exercise on telomere length in the ventral hippocampus (VH) and prefrontal cortex (PFC) of the rat. Forty-five male Sprague–Dawley rats were categorised into four groups: maternally separated runners (MSR), maternally separated non-runners (MSnR), non-maternally separated runners (nMSR) and non-maternally separated non-runners (nMSnR). Behavioural analyses were conducted to assess anxiety-like behaviour and memory performance in the rats, after which relative telomere length was measured using qPCR.

**Results:**

Maternally separated (MS) rats exhibited no significant differences in either anxiety levels or memory performance on the elevated-plus maze and the open field compared to non-maternally separated rats at 49 days of age. Exercised rats displayed increased levels of anxiety on the day that they were removed from the cages with attached running wheels, as well as improved spatial learning and temporal recognition memory compared to non-exercised rats. Exploratory post-hoc analyses revealed that maternally separated non-exercised rats exhibited significantly longer telomere length in the VH compared to those who were not maternally separated; however, exercise appeared to cancel this effect since there was no difference in VH telomere length between maternally separated and non-maternally separated runners.

**Conclusions:**

The increased telomere length in the VH of maternally separated non-exercised rats may be indicative of reduced cellular proliferation, which could, in turn, indicate hippocampal dysfunction. This effect on telomere length was not observed in exercised rats, indicating that voluntary exercise may buffer against the progressive changes in telomere length caused by alterations in maternal care early in life. In future, larger sample sizes will be needed to validate results obtained in the present study and obtain a more accurate representation of the effect that psychological stress and voluntary exercise have on telomere length.

## Background

The development of human physiology and behaviour is a gradual process, which is constantly influenced by the intricate interplay between genetic and environmental factors. In terms of neurodevelopment, the neonatal period is marked by continuous proliferation and maturation of synapses and neurotransmitter pathways [1], rendering the brain sensitive to internal and external stimuli throughout this time. Exposure to adverse experiences during early childhood may therefore have a long-lasting, debilitating impact on several aspects of physiological and psychological development [2,3], ultimately unfolding in anxiety disorders including posttraumatic stress disorder (PTSD).

Although the exact mechanisms through which stress influences physical and psychological wellbeing are presently unclear, increasing evidence supports the involvement of accelerated cell aging in this process [4]. The extent of this progressive aging in eukaryotic organisms can be catalogued by means of telomeres, the guanine-rich sections of recurring DNA sequences at the extremities of chromosomes [4-6]. During mitotic cell division, the 3’ ends of linear chromosomes are not fully replicated by DNA polymerase [7], resulting in their progressive shortening through the course of each replication cycle [8]. Telomeres play an integral role in maintaining genomic stability and preventing chromosomal end-fusion [9] by enabling replication without the loss of nucleotide bases, and thus genetic information, at the ends of DNA strands. In stem cells and adult germ cells, the erosion of telomeric regions is normally compensated for through the action of telomerase, which inserts essential telomeric nucleotides onto the ends of these structures [10]. However, telomerase activity is virtually undetectable in human and mouse somatic cells [11,12], and for the most part, telomeres in these cells remain shortened. In the event of significant telomere attrition, cell proliferation ceases, leading to a state of senescence or apoptosis [13]. Telomere length (TL) can therefore serve as an indicator of a cell’s biological age [4] and predict its future proliferative potential [6].

To a large extent, TL is governed by genetic factors [9], with heritability estimates obtained from family and twin studies ranging between 78% and 82% [14,15]. In addition, a number of lifestyle factors, such as smoking, sleep duration, body mass index (BMI) and physical activity can alter telomere length [16,17]. Shorter TL has been associated with an overall detrimental effect on cardiovascular health [16,18-20] cancer and a spectrum of age-related disorders [21]. In addition, self-perceived psychological stress, as well as the chronicity of stress has been linked to shorter TL in humans [4,6]. Similarly, shorter blood leukocyte TL has been reported in caregivers who experience high levels of stress [22] and in patients with mood disorders [23].

Exposure to adverse experiences such as psychological stress may have the greatest neurobiological impact during early development, when the rate of telomere shortening is at its peak and factors involved in TL regulation may be encoded [24,25]. Childhood physical and emotional maltreatment has been associated with increased blood leukocyte telomere attrition in psychiatrically healthy adults [26], as well as in individuals with anxiety disorders compared to control individuals [27]. Likewise, shorter leukocyte TL was observed in female mice that were subjected to reproductive stress and their male counterparts exposed to crowding stress, compared to control groups [5].

Not all individuals who experience stress develop chronic pathology, and likewise, not all of these individuals will display shortened DNA telomeric regions. This observation highlights the possibility of supplementary mechanisms that may remediate the effects of stress on telomere shortening. One such approach is physical exercise, which has been linked to longer leukocyte TL, in both trained athletes and a general sample of twins [17,28] Puterman et al. [29] found shorter blood leukocyte TL to be associated with higher levels of perceived stress in non-exercised individuals, whereas this effect seemed to be ameliorated in exercised individuals, thereby supporting the hypothesis that physical exercise may serve as a buffer for the detrimental effects of perceived psychological stress.

TL may serve a role as a biomarker of subsequent stress responses in stress-exposed individuals. Since previous studies have mainly measured TL in peripheral blood mononuclear cells, we sought to investigate the impact of early developmental stress and voluntary exercise on relative telomere length (RTL) in tissues relevant to this process, namely the rat ventral hippocampus (VH) and prefrontal cortex (PFC).

## Results

### Behavioural analyses

The results of the behavioural studies have been reported previously [30]. There were no significant differences in anxiety levels, as measured in the open field (OF) and elevated plus maze (EPM), in maternally separated (MS) and non-maternally separated (nMS) rats at postnatal day 49 (PND49). In contrast, exercised rats (runners, R) exhibited increased levels of anxiety-like behaviour in both of these tests on the day that they were removed from their cages with attached running wheels. Similarly, maternal separation did not produce any change in memory performance. Exercised rats (MSR and nMSR) performed significantly better in the Morris water maze (MWM) and the temporal order task in comparison to non-exercised rats (MSnR and nMSnR).

### Relative telomere length (RTL)

Slopes of the standard curves plotted from the mean C_q_ versus the log of serial dilution concentrations ranged from -2.26 to -3.31, yielding corresponding PCR efficiencies between 100% and 130% (Table 1). The correlation coefficients for all assays were greater than the generally accepted 0.985. Six of the 44 samples from the VH assays and 9 of the 43 PFC samples were excluded from further analysis due to non-amplification, C_q_ values exceeding 30 or differences in C_q_ values between two or more of the triplicates being greater than 0.5.

**Table 1:** **PCR efficiencies and correlation coefficients for Tel**_**1**_**and AT**_**1**_**assays of the left VH and left PFC**

**Brain region**	**Assay**	**Standard curve slope**	**PCR efficiency**	**R**^**2**^
**VH**	Tel_1_	-2.86	123%	0.998
	AT_1_	-3.32	100%	0.986
**PFC**	Tel_1_	-2.76	130%	0.994
	AT_1_	-3.30	100%	0.995

VH, ventral hippocampus; PFC, prefrontal cortex; R^2^, correlation coefficient.

**Figure 1:**
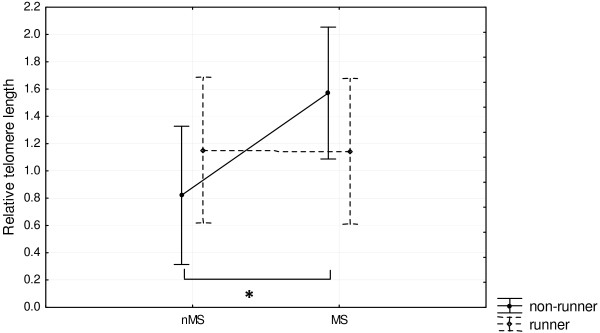
**The effect of maternal separation and voluntary exercise on relative telomere length (RTL) in the left ventral hippocampus.** *RTL was significantly longer in MSnR compared to nMSnR (p = 0.037). Data represent means ± SEM. Abbreviations: MSR, maternally separated exercised rats; MSnR, maternally separated non-exercised rats; nMSR, non-maternally separated exercised rats; nMSnR, non-maternally separated non-exercised rats

Some deviations from the analysis of variance (ANOVA) assumptions were detected; however, further analysis done on log-transformed data yielded essentially the same results. Results for the untransformed data are thus reported. No significant variance was detected in the PFC tissues. In the VH, the two-way ANOVA did not reveal significant effects of stress or exercise (F_1,32_ = 2.24, p = 0.14) (Figure 1 and Table 2). However, although the interaction was not significant, post-hoc Fisher’s least significant difference (LSD) test revealed that the RTL was significantly longer in the MSnR group in comparison to the nMSnR group (p = 0.037). A non-parametric bootstrap test with Bonferroni post-hoc correction confirmed the trends reported. RTL did not differ significantly when MSR were compared to nMSR, when MSR were compared to MSnR or when nMSR were compared to nMSnR.

**Table 2:** Descriptive data pertaining to the effect of maternal separation and voluntary exercise on RTL in the left ventral hippocampus

**Experimental group**	**n**	**Mean RTL**	**SEM**	**95% CI**
nMSnR	10	0.821	0.061	0.682–0.960
nMSR	9	1.152	0.101	0.919–1.385
MSnR	11	1.571	0.397	0.687–2.454
MSR	9	1.142	0.219	0.638–1.647

Abbreviations: nMSnR, non-maternally separated non-runners; nMSR, non-maternally separated runners; MSnR, maternally separated non-runners; MSR, maternally separated runners; RTL, relative telomere length; SEM, standard error of means; CI, confidence interval.

Two-way ANOVA revealed that there was no significant effect of stress or voluntary exercise on the RTL in the PFC. Furthermore, the interaction was not significant, and RTL in the PFC did not differ significantly between any of the experimental groups.

## Discussion

Maternal separation paradigms have been used extensively to investigate the impact of early-life adversity, and have been shown to have numerous long-term consequences, including increased levels of anxiety-like behaviour and neurochemical changes, pointing towards HPA axis dysregulation and impaired memory formation [31-33]. However, the maternally separated rats used in the present study did not display increased anxiety-like behaviour or memory impairment [30]. In terms of anxiety, several authors have failed to detect significant effects of repeated maternal separation [31-36]. This may be a consequence of upregulation of maternal care and increased arched-back nursing, licking and grooming of pups by the dam when they are returned to the cage, which may counteract the stressful effects of maternal separation [37,38]. The inconsistent results may also be due to the use of highly variable maternal separation paradigms between studies [39]. In addition, it is possible that anxiety-like behaviour is not readily observed in adolescence (PND49). To this end, in a separate study, rats subjected to the same maternal separation protocol exhibited anxiety-like behaviour in adulthood (PND75) [40].

Exercised rats used in the present study displayed significantly increased levels of anxiety compared to sedentary controls [30]. This was, however, attributed to the stress of being removed from the running wheels on the same day that the OF and EPM tests were performed [30].

Although no effects of stress or exercise on RTL were observed in the present study, an exploratory post-hoc analysis revealed a significant difference in RTL in the VH between rats in the MSnR and nMSnR groups, while the RTLs of the MSR and nMSR were virtually indistinguishable. MSnR rats were found to possess significantly longer telomeres when compared to nMSnR rats. These results are interesting in light of those obtained by Hulshof et al. [41] who found that, although the maternal separation protocol resulted in no overt differences in anxiety-like behaviour between maternally separated and control Wistar rats, maternally separated rats exhibited significantly reduced cell proliferation in the VH, which the authors suggest could interfere with the normal development of the hippocampus. The results from Hulshof et al. [41] corroborated those obtained in previous studies [42,43]. Telomeres reduce in length with each cell division; therefore, the lower the proliferative capabilities of the tissue, the longer the telomeres will be. The increase in RTL in MSnR in the present study may thus be indicative of reduced cellular proliferation in the VH of those rats, which could indicate alterations in normal development of this brain region. Although the functional implications of reduced proliferative ability in the VH are currently unknown, the results suggest that voluntary exercise could serve as a buffer against the progressive changes in RTL caused by alterations in maternal care early in life. One way in which exercise may buffer changes in RTL is by moderating the overall burden of oxidative stress on the body, and specifically in the VH, through the upregulation of genes that encode powerful antioxidant enzymes [44]. Indeed, regular, moderate exercise has been shown to increase the body’s endogenous antioxidant activity and its resistance to oxidation [45].

No further significant differences were detected as a result of maternal separation or exercise in either the VH or PFC. These results contrast with data from previous studies indicating that psychological stress gives rise to shorter TL in human peripheral blood mononuclear cells and leukocytes [4-6,26,46]. However, it is important to note that the vast majority of studies associating shorter TL with stress have been conducted in humans, utilising DNA extracted from peripheral blood mononuclear cells instead of brain tissue. Telomere length has been found to vary in a tissue-specific fashion [47,48]. For example, Thomas et al. [49] reported shorter TL in white blood cells and buccal cells, but longer TL in the hippocampi of patients with Alzheimer’s disease, compared to control individuals. In addition, results from a recent study indicate that chronic exercise alters TL in a tissue-specific manner [50].

Although multiple factors, including physiology, nutrition, reproductive ability, behaviour and strain should be taken into account, it is generally accepted that most rats reach maturity at 40 to 60 days of age [51]. It has been proposed that differences in TL might be age-related, with significant variation only presenting in older cohorts [6]. Since the normal lifespan of rats ranges from two-and-a-half to three years, more distinct differences in RTL might become apparent at a later age. Given that altered TL has been previously associated with both the severity and chronicity of psychological stress [4], as well as the number of adverse early life events in individuals suffering from anxiety disorders [46], stress is likely to have additive and multiplicative effects on RTL.

## Conclusions

The significant results in the present study, although exploratory, are interesting and as such, warrant further investigation in a larger sample. Future studies will benefit from measurements of telomerase activity and markers of oxidative stress, in addition to measurements of RTL in a larger sample group, in order to obtain a more accurate representation of the effect that developmental stress and voluntary exercise might have on RTL.

## Methods

### Ethical approval

All animal experiments were approved by the University of Cape Town’s Faculty of Health Sciences Research Ethics Committee. Animal experiments were conducted in accordance with the South African National Guidelines, namely ‘The South African National Standard: the care and use of animals for scientific purposes’. The rats were anaesthetised by exposure to halothane vapour (Lakato (Pty) Ltd, Cape Town, South Africa) immediately prior to decapitation.

### Behavioural analyses

Forty-five male Sprague–Dawley (*Rattus norvegicus*) rat pups were housed in culled groups of eight, together with their dams, under standard conditions [30]. Food and water was available *ad libitum*. Maternal separation was performed as previously described. Briefly, on PND2, rat pups were randomly assigned to one of four experimental groups as follows: maternally separated runners (MSR; n = 10), maternally separated non-runners (MSnR; n = 11), non-maternally separated runners (nMSR; n = 11) and non-maternally separated non-runners (nMSnR; n = 13). On PND2, the dams of pups in the maternally separated group were removed from their home cages and the pups were transferred, in their home cages, to another room in order to prevent communication by ultrasound vocalizations. After 3 hours, the dams were reunited with their pups in the home cage. This protocol was repeated once a day between 9:00 and 13:00 until PND14, after which normal interaction between the dam and her pups resumed.

Pups were weaned on PND21, and voluntary exercise was initiated on PND29 by placing rats (MSR and nMSR) in individual cages with running wheels attached. Non-exercised rats (MSnR and nMSnR) were housed singly in standard plastic cages from PND29 to PND49. Exercised rats were removed from the cages with attached running wheels on PND49 and housed in standard cages in groups of twos or threes until termination of the experiment.

Anxiety-like behaviour was appraised in the OF and EPM at PND49, as described by Grace et al. [30]. The MWM and several object recognition tasks were used to assess memory performance from PND50 to PND64, as previously described [30].

### DNA isolation and preparation

Rats were decapitated on PND65, after which the VH and PFC were dissected from the left hemisphere of each rat and stored at -80°C in RNAlater® solution. Genomic DNA was purified from these regions using an AllPrep DNA/RNA Mini Kit (Qiagen, Hilden, Germany), with slight modifications made to the standard protocol. The concentration and quality of the DNA were verified by means of the Nanodrop® ND-1000 spectrophotometer (Nanodrop Technologies, Wilmington, DE, USA). Separate calibrator samples consisting of pooled DNA from 44 VH and 43 PFC samples respectively were created and serially diluted to yield eight standards with concentrations ranging from 50 ng/ul to 1.32 ng/ul.

### Quantitative real-time PCR

Amplification of telomeric DNA (T), together with that of the single-copy genomic *AT*_*1*_ receptor (S) was performed on a MicroAmp Optical 384-well Reaction Plate (Applied Biosystems, Foster City, CA, USA) using Applied Biosystem’s 7900HT Fast Real-Time polymerase chain reaction (qPCR) system and the slightly modified protocol of Cawthon et al., 2002 [52]. Plates were prepared with the epMotion 5070 (Eppendorf, Hamburg, Germany) so that each 10 μl reaction mixture for the telomere reaction (*Tel*_*1*_) for both VH and PFC samples consisted of 5 μl QuantiFast 2 × SYBR Green PCR mastermix (Qiagen, Hilden, Germany), 270 nM forward primer (5’-*GGTTTTTGAGGGTGAGGGTGAGGGTGAGGGTGAGGGT*-3’), 900 nM reverse primer (5’-*TCCCGACTATCCCTATCCCTATCCCTATCCCTATCCCTA*-3’), 25 ng genomic DNA and water. The specific reaction mix for *AT*_*1*_ was similar except that it included 800 nM forward primer (5’-*ACGTGTTCTCAGCATCGACCGCTACC*-3’) and 600 nM reverse primer (5’-*AGAATGATAAGGAAAGGGAACAAGAAGCCC*-3’) for the VH samples, and 700 nM and 500 nM of forward and reverse primer respectively for the PFC samples. All samples were run in triplicate in order to account for possible technical variation. Cycling conditions were as follows: 95°C for 5 minutes, followed by 40 cycles of 95°C for 10 seconds and 54°C for 30 seconds in the case of the VH and PFC *Tel*_*1*_ and PFC *AT*_*1*_ assays, or 60°C in the case of the VH *AT*_*1*_ assay.

Rigorous control measures were put in place to ensure the quality of the data. An additional melting curve analysis consisting of 95°C for 15 seconds, 60°C for 15 seconds and 95°C for 15 seconds was performed at the end of each reaction to verify specific amplification. The serial dilution series was used in standard curve construction, and all quantification cycle (C_q_) values were corrected according to efficiencies calculated from the standard curve. Threshold and baseline values were automatically determined by the Sequence Detector Systems (SDS) version 2.4 software. Samples with a difference in C_q_ values greater than 0.5 between triplicates were disregarded in further analyses.

### Data analysis

The RTL was determined as the factor by which the experimental sample differed from the reference sample in its ratio of telomere repeat copy number (T) to the *AT*_1_ copy number (S) [52]. A two-way ANOVA was applied to the data, followed by post-hoc Fisher’s LSD tests. A non-parametric bootstrap test with Bonferroni post-hoc correction was performed to eliminate the possibility of unequal variances. Statistically significant results with p values of less than 0.05 are reported.

## Abbreviations

TL: Telomere length; RTL: Relative telomere length; VH: Ventral hippocampus; PFC: Prefrontal cortex; PND: Postnatal day; MSR: Maternally separated runners; MSnR: Maternally separated non-runners; nMSR: Non-maternally separated runners; nMSnR: Non-maternally separated non-runners; OF: Open field; EPM: Elevated-plus maze; MWM: Morris water maze.

## Competing interest

The authors declare that they have no competing interests.

## Authors’ contributions

MB carried out the molecular genetic studies and drafted the initial manuscript. LG carried out the behavioural analyses and assisted with drafting the manuscript. KB carried out behavioural analyses and participated in the design of the study. VR conceived of the behavioural study and assisted in drafting the manuscript. MK performed the statistical analyses. SS participated in the design and coordination of the study and assisted with drafting the manuscript. SH conceived of the idea, participated in coordination of the design of the study and assisted in drafting the manuscript. All authors read and approved the final manuscript.
